# Pooled Analysis of the Efficacy and Safety of Video Capsule Endoscopy in Patients with Implantable Cardiac Devices

**DOI:** 10.1155/2019/3953807

**Published:** 2019-05-19

**Authors:** Rabih Tabet, Najib Nassani, Boutros Karam, Youssef Shammaa, Philippe Akhrass, Liliane Deeb

**Affiliations:** ^1^Department of Internal Medicine at Staten Island University Hospital, Northwell Health, New York, USA; ^2^Division of Gastroenterology & Hepatology at University of Illinois at Chicago, Chicago, USA; ^3^Division of Cardiology & Electrophysiology at Staten Island University Hospital, Northwell Health, New York, USA; ^4^Division of Gastroenterology & Hepatology at Staten Island University Hospital, Northwell Health, New York, USA

## Abstract

**Background:**

To date, video capsule endoscopy (VCE) is still contraindicated by the FDA and the main manufacturers of Cardiac Implantable Electronic Devices (CIED) in patients with CIED, given a theoretical electromagnetic interference and possible device malfunction.

**Objectives:**

The objective of this study was to assess the safety profile and efficacy of VCE in patients with implantable cardiac devices through analyzing the risk of mutual interference.

**Methods:**

A systematic review of PubMed, Web of Science, and Embase databases was conducted. Peer-reviewed original articles, published in the English language and containing “capsule endoscopy” AND “pacemaker”, “defibrillator” OR “left ventricular assist device” as keywords, were selected. Studies performed in vitro, isolated case reports, and abstracts/posters were excluded.

**Results:**

A total of 735 VCE procedures were performed in patients with cardiac devices in various clinical settings. Cardiac events were not seen in any case. Interference on capsule images transmission was noted in 5 cases (left ventricular assist device (LVAD)) where few images were lost when the capsule was closest to the device. Finally, interference between capsule and telemetry leads was noted in 6 cases (4 Permanent Pacemakers (PPM), 2 Implantable Cardioverter-Defibrillator (ICD)) leading to image artifacts.

**Discussion:**

Adverse cardiac events were not seen in any study. Loss of images occurred when the VCE was in proximity to the device (only with LVAD) or after telemetry leads installation without affecting the completion rate and diagnostic yield of VCE.

**Conclusion:**

VCE is safe and remains efficient in patients with cardiac devices. If cardiac monitoring is required, wired systems are preferable.

## 1. Introduction

Gastrointestinal bleeding that persists or recurs without any obvious etiology after performing an upper endoscopy and colonoscopy originates from the small bowel (SB) in the majority of cases [1]. The gold standard test for suspected SB bleeding is video capsule endoscopy (VCE). It was first introduced into clinical practice in 2001 and has emerged given its noninvasiveness and effectiveness as it allows the examination of the entire length of the SB with high quality image acquisition. It is contraindicated in patients with suspected partial or intermittent SB obstruction and pregnant women. A significant proportion of patients presenting with suspected SB bleed have implantable cardiac devices. The use of VCE in this population has been subject to debate given the possible electromagnetic interference. In 2009, the European Society of Gastrointestinal Endoscopy (ESGE) recommended the use of VCE in patients with Permanent Pacemakers (PPM) and Implantable Cardioverter-Defibrillators (ICD) [2]. However, VCE in patients with PPM or ICD is still contraindicated according to the last US Food and Drug Administration (FDA) update issued on November 28, 2001, [3] and to the main manufacturers recommendations.

## 2. Video Capsule Endoscopy System

Video Capsule Endoscopy is performed using an ingestible imaging capsule that passes naturally through the gastrointestinal tract, capturing and transmitting video images along its way, to an array of electrodes affixed to the patient's thorax. These electrodes detect data signals and send them directly to an external recording device, which collects and stores the video images to be downloaded to a computer workstation for review and diagnosis. The available capsules on the market are PillCam™ SB (Given Imaging Ltd), EndoCapsule™ (Olympus), MicroCam™ (IntroMedic), and OMOM™ small-bowel capsule (Chongqing Jinshan Science and Technology Group).

## 3. Electromagnetic Interference

The implantation of PPM and ICD for the treatment of brady- and tachy-arrhythmia has increased significantly over the last 30 years [4]. Likewise, there has been an increase in the development and use of technology that emits electromagnetic radiations that may disturb the behavior of cardiac implantable electronic devices (CIED) with potential harmful consequences. Because one of the most essential components of CIED function is accurate sensing of intrinsic cardiac potentials, any interference by nonphysiologic sources may cause the system to interpret such interference as cardiac in nature [4]. This noise sensing can have drastic adverse clinical consequences, such as the failure to pace when needed, or the triggering of ICD therapies due to inappropriate identification of noise as ventricular tachycardia or ventricular fibrillation [4]. This interference between two separate electronic devices is called electromagnetic interference (EMI). The sources of EMI can be related to every-day-use devices like cellular phones and metal detectors [4] or can be iatrogenic like radiation therapy, electrocautery, transcutaneous electric nerve stimulation, electroconvulsive therapy, and magnetic resonance imaging [4]. Video capsule generates an electromagnetic field and theoretically can be a source of EMI with implantable cardiac devices having possible consequences on patients.

We performed this pooled analysis of the published literature in order to investigate the efficacy and safety of VCE in patients with implantable cardiac devices including Permanent Pacemakers (PPM), Implantable Cardioverter-Defibrillators (ICD) and Left Ventricular Assist Devices (LVAD).

## 4. Materials and Methods

**Figure 1:**
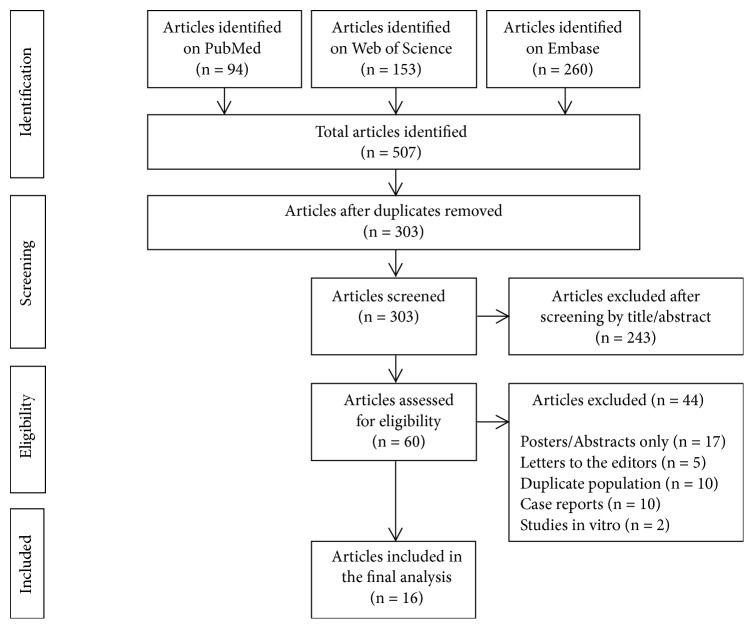
PRISMA diagram showing the selection process of the articles included in the final analysis.

Articles published in English language on VCE and implantable cardiac devices from 1/1/04 till 9/1/17 were searched through PubMed, Web of Science, and Embase databases using the keywords (MeSH terms): “video capsule endoscopy” OR “capsule endoscopy” OR “wireless capsule endoscopy” OR “wireless capsule endoscope” OR “capsule endoscope” OR “video capsule endoscope” OR “video capsule”; combined with either: “heart devices”, “pacemaker”; “defibrillator”, “assist device”, or “heart” AND “Interference”. The title and abstracts of the retrieved publications were reviewed and screened initially. Then, data selection occurred after manuscript review. Studies focusing on capsule endoscopy used in conjunction with the presence of cardiac implantable device: pacemaker and/or defibrillator and/or left ventricular assist device* in vivo* were analyzed. In the case of duplicate studies, the most recent study including the latest series of patients was retained. Isolated case reports, letters to the editors, and review articles were excluded (Figure 1).

Data on patients' demographics, indications for video capsule endoscopy, technical complications, and diagnostic and completion rates were collected. The main outcome was the safety of the VCE procedure defined as the successful completion of video capsule endoscopy without cardiac adverse events or cardiac device malfunction. Cardiac adverse events included arrhythmias during VCE and a change in the cardiac device settings after VCE. Secondary outcome included the quality of the transmitted images reflecting interference of the implantable cardiac devices with the VCE, completion rate and diagnostic rate. Data was analyzed using SPSS software.

## 5. Results

**Figure 2:**
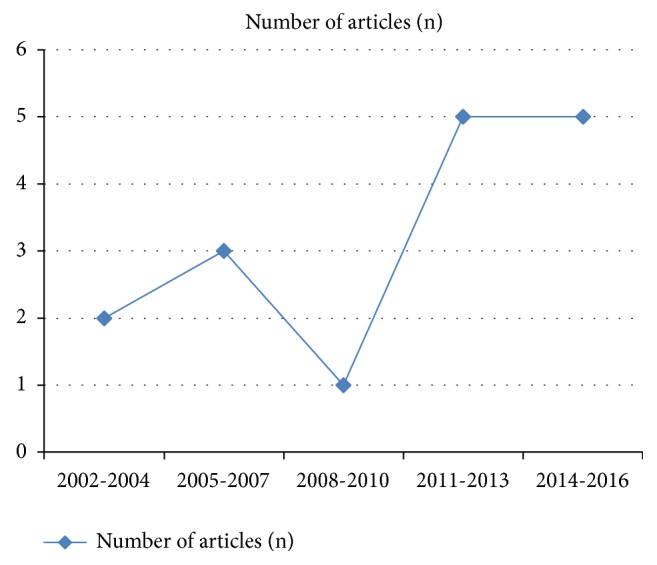
Number of published articles by year showing increasing interest in this topic.

A total of 16 original articles (10 retrospective and 6 prospective studies) [5–20], involving a total of 735 VCE procedures, were included in our analysis. The selected publications were sorted per year. The number of publications per year has notably increased from 2011 through 2016 (Figure 2).

This increase in the number of studies might correlate with the publication of the recommendations by the European Society of Gastrointestinal Endoscopy (ESGE) in 2009 allowing more patients with implantable cardiac devices to get VCE procedures and thus increasing the studied population number and studies.

### 5.1. Baseline Characteristics

Table 1 recapitulates the 16 studies included in the final analysis along with the baseline characteristics of the population. The majority of VCE procedures were performed in patients with PPM (487 out of 735 procedures), followed by ICDs (162 procedures). 86 of the 735 procedures were performed in patient with LVADs. The most common PPM used was Medtronic™ (43% of all PPM) followed by Guidant™ (30% of all PPM). Among ICDs, Medtronic™ (31%) was primarily used, followed by St Jude™ (27%) and Guidant™ (21%). Finally, 92% of the LVAD studied were Thoratec HeartMate II™. The three main capsules used in the selected studies were PillCam™ (93%), EndoCapsule™ (6%), and MiroCam™ (1%). The OMOM™ capsule was not studied in any of the selected publications. The pooled median age of the included patients was 69.8 years with 64% of males and 36% of females. The most common indication for VCE was gastrointestinal bleeding (76%) including both overt and occult bleeding. In 20% of patients the indication for VCE was iron deficiency anemia. The remaining (4%) had various other indications such as chronic abdominal pain, celiac disease, Peutz-Jeghers disease, and Crohn's disease. The pooled completion rate of VCE in patients with cardiac devices was 85% and the diagnostic yield of VCE in this population was 70%.

### 5.2. Cardiac Outcome and Quality of the Transmitted Images

The pooled adverse cardiac events rate was 0%, as well as the pooled cardiac device malfunction rate. No oversensing by PPMs or delivery of inappropriate shock by ICDs was noted. Supraventricular and/or ventricular premature contraction occurred in two patients without any clinical symptom. These two patients had similar arrhythmias detected on their baseline electrocardiogram prior to VCE. In one case, the video capsule showed low signal resulting in a transient lack of localization of the capsule without any loss of images (Video capsule: PillCam™; PPM: ELA Sorin™). Some impairment of the quality of VCE images consisting of artifacts and/or recording gaps occurred in 6 patients (4 PPM and 2 ICD, 0.8% overall) and this happened only after the installation of the telemetry leads. Finally, artifacts in the capsule images occurred in five patients with LVAD (0.6% overall; 5.8% among patients with LVADs) when the capsule was in the upper abdomen without any cardiac device malfunction or arrhythmia occurrence.  Table 2 summarizes the VCE procedures, cardiac outcome, and quality of the transmitted images.

## 6. Discussion

The feasibility and safety of wireless video capsule endoscopy in patients with cardiac devices have been called into doubt since its introduction in 2001 by extrapolation to the early negative experiences of cardiac device malfunction with magnetic resonance imaging and electrocautery current. However, the benefits of identifying the source of bleed in patients with cardiac devices are not to be ignored. Especially, patients with cardiac devices mostly have advanced heart disease, dilated cardiomyopathies, and coronary artery disease making of them the candidates par excellence to receive antiplatelets and/or anticoagulants and to be at risk for gastrointestinal bleeding and undergo the ensuing work-up. There has been a growing interest in assessing the safety and feasibility of VCE in this population that was reflected by the increasing number of clinical studies over the recent years. Theoretically, electromagnetic interference between both devices is possible and its impact on either of the devices is yet to be determined [4]. However, interference with the cardiac devices is not an expected event physically, given that the signal magnitude from the capsule recorder is too low to interfere with PPMs/ICDs, in addition to several safety mechanisms that isolate most of the manufactured pacemakers/defibrillators from their surroundings such as the hermetically sealed titanium housing around the device, the bipolar mode programming, and the provision of filters in the amplifier [4].

Following initial reports of uneventful VCE in patients with cardiac devices, many centers worldwide started performing VCE in this population. Various types and brands of cardiac devices were used in combination with different brands of video capsules. The most commonly used capsules and heart devices suggesting a more solid evidence of safety were the PillCam™ (Given Imaging) and EndoCapsule™ (Olympus) with PPMs and ICDs from Medtronic™, St Jude™, and Guidant™ and Thoratec HeartMate II™ LVAD. This can be nothing but the reflection of the earlier introduction of these brands to the market than the other studied capsules and devices chronologically.

In an effort to address the safety profile, our pooled analysis of clinical studies did not detect any serious adverse cardiac events. In two cases, atrial and ventricular premature contractions were observed without any clinical significance or impact. In fact, these two patients had similar arrhythmias detected on their baseline electrocardiograms prior to VCE.

The most common indication for VCE in our select population of patients with implantable cardiac devices was obscure gastrointestinal bleeding (76%). This proportion is comparable to the results found by Liao et al. (2010) who performed a pooled analysis of 22,840 small-bowel capsule endoscopy procedures for the evaluation of patients with small-bowel signs and symptoms, with the most common indication being obscure GIB (66%) [21]. The pooled completion rate of VCE in our patient population was 85% and the detection rate of VCE was 70% comparable to the overall pooled completion rate of 83.5% (83.6% for OGIB) and a pooled detection rate of 59.4% overall (60.5% for OGIB) in the studied general population of Liao et al. (2010), respectively [21].

Some interference has been observed in image transmission leading to the loss of few images when the VCE was in proximity to the device (in the distal esophagus and stomach mainly) or after telemetry leads installation with a pooled “interference with image transmission” of 1.4% overall. In half of these cases, the interference with image transmission was related to the telemetry leads rather than the cardiac device per se. It is worth pointing out that this low interference with image transmission did not affect the completion rate or the diagnostic yield of the isolated nor overall procedures and this was reflected by the comparable rates of completion and detection to the general population as mentioned previously [21]. Given that interference was noted in isolated cases between VCE and telemetry systems, a wired system of monitoring would be preferable if cardiac monitoring is required by the local policy of the center or at the discretion of the physician.

Another observation is worth noting concerning the MicroCam™ (IntroMedic) which uses electric-field propagation system to transmit the images instead of the traditional radiofrequency signal transmission. Chung et al. (2012) prospectively studied this brand of video capsule in 6 patients (3 PPM and 3 ICD) [12]. It was suggested that even with the human body communication technique, data transmission was safe and effective. No disturbances in PPM or ICD function were identified and the images transmitted remained of good quality [12]. Nevertheless, the data on the MicroCam™ remains much less solid compared to the PillCam™ and EndoCapsule™ because of the very small number of procedures (1% of the total VCE) performed with this brand of capsule.

Several limitations might have hurdled this pooled analysis, mainly the low power of isolated studies and the retrospective design and observational nature of the analyzed studies. Also, the myriad of brands and types of cardiac devices and capsules limit the generalization of the results. However, this study represents the first pooled analysis on the subject reflecting clinical data and providing evidence of safe and efficient use of VCE in this disadvantaged population. Solid evidence does not exist to undermine the use of diagnostic means guiding further management and possible outcome in patients with cardiac devices undergoing video capsule endoscopy.

## 7. Conclusion

According to available data, VCE can be performed safely and efficiently in patients with cardiac devices. Nevertheless, electromagnetic interference can occur between VCE and LVADs and with wireless telemetry leads causing impairment of the recordings and leading to some artifacts and gaps but without affecting the diagnostic yield of the procedure or the cardiac safety of patients. If cardiac monitoring is required, wired systems are preferable.

## Figures and Tables

**Table 1:** Studies included in the analysis and the baseline characteristics of the patients.

Study	Year	n	Mean age	Male	Female	Completion rate	Diagnostic rate
Leighton [5]	2004	5	73	4/5 (80%)	1/5 (20%)	5/5 (100%)	n/a
Payeras [6]	2005	20	78	14/20 (70%)	6/20 (30%)	20/20 (100%)	14/20 (70%)
Dubner [7]	2005	6	61	4/6 (66%)	2/6 (34%)	n/a	n/a
Leighton [8]	2005	5	72	1/5 (20%)	4/5 (80%)	n/a	n/a
Dirks [9]	2008	5	45	0/5 (0%)	5/5 (100%)	5/5 (100%)	5/5 (100%)
Elias [10]	2009	4	78	2/4 (50%)	2/4 (50%)	n/a	n/a
Bandorski [11]	2011	59	73	n/a	n/a	n/a	n/a
Chung [12]	2012	6	60.5	3/6 (50%)	3/6 (50%)	6/6 (100%)	n/a
Bandorski [13]	2012	380	n/a	n/a	n/a	n/a	n/a
Cuschieri [14]	2012	20	71	13/20 (65%)	7/20 (35%)	n/a	n/a
Harris [15]	2013	118	72	74/118 (63%)	44/118 (37%)	79/118 (67%)	n/a
Stanish [16]	2014	21	71.1	8/21 (38%)	13/21 (62%)	20/21 (95%)	13/21 (62%)
Moneghini [17]	2016	14	66.4	11/14 (78%)	3/14 (22%)	13/14 (92.8%)	9/14 (64.2%)
Amornsawadwattana [18]	2016	30	60.1	24/30 (80%)	6/30 (20%)	30/30 (100%)	12/30 (40%)
Truss [19]	2016	8	63	8/8 (100%)	0/8 (0%)	8/8 (100%)	8/8 (100%)
Hanson [20]	2016	34	67.1	n/a	n/a	34/34 (100%)	31/34 (91%)
*Pooled numbers*		*735*	*69.8*	*166/262 (64*%)	*96/262 (36*%)	*220/261 (85*%)	*92/132 (70*%)

**Table 2:** Pooled results of the 16 studies included in the analysis [5–20].

Type of Device	Total Number of cardiac devices (n)	Cardiac devices brand	VCE brand	Cardiac Outcome	Quality of transmitted images
PPM	487	Medtronic (77) Guidant (54) St Jude Medical (26) Biotronik (12) Vitatron (8) ELA Sorin (3) Boston scientific (1) No specification (306)	PillCam (431) EndoCapsule (33) MiroCam (3) No specification (20)	No adverse cardiac events No cardiac device malfunction 1 case had some atrial and ventricular premature contractions without clinical symptoms	4 cases of VCE interference with telemetry leads leading to few images loss 1 case showed low capsule signal resulting in transient lack of localization of the capsule without images loss (PillCam with ELA Sorin)

ICD	162	Medtronic (15) St Jude (13) Guidant (10) Boston scientific (7) ELA Sorin (2) Biotronik (1) No specification (114)	PillCam (147) EndoCapsule (8) MiroCam (3) No specification (4)	No adverse cardiac events No cardiac device malfunction 1 case had some atrial and ventricular premature contractions without clinical symptoms	2 cases of VCE interference with telemetry leads leading to few images loss

LVAD	86	Thoratec HeartMate II (35) HeartWare (3) No specification (48)	PillCam (52) No specification (34)	No adverse cardiac events No cardiac device malfunction	5 cases of possible interference leading to images loss/artifact when VCE was near the device

Permanent Pacemaker (PPM); Implantable Cardiac Defibrillator (ICD); Left Ventricular Assist Device (LVAD); Video Capsule Endoscopy (VCE).

## Abbreviations

VCE: Video Capsule Endoscopy
PPM: Permanent Pacemaker
ICD: Implantable Cardioverter Defibrillator
LVAD: Left Ventricular Assist Device
SB: Small Bowel
CIED: Cardiac Implantable Electronic Device
EMI: Electromagnetic Interference.
